# Airway inflammatory profile among cleaning workers from different workplaces

**DOI:** 10.1186/s12890-022-01949-5

**Published:** 2022-04-29

**Authors:** Edinéia Rosa da Paz, Cynthia Mafra Fonseca de Lima, Soraia Nogueira Felix, Bruna Schaeffer, Clóvis Eduardo Santos Galvão, Aristides Tadeu Correia, Renato Fraga Righetti, Milton de Arruda Martins, Iolanda de Fátima Lopes Calvo Tibério, Beatriz Mangueira Saraiva-Romanholo

**Affiliations:** 1 Instituto de Assistência Médica Ao Servidor Público Estadual (IAMSPE), Hospital Do Servidor Público Do Estado de São Paulo, Sao Paulo, SP Brazil; 2 Disciplina de Imunologia Clínica e Alergia, HCFMUSP, Universidade de São Paulo, Sao Paulo, SP Brazil; 3 Universidade Cidade de São Paulo (UNICID), São Paulo, SP Brazil; 4 Departamento de Imunologia Clínica e Alergia, HCFMUSP, Universidade de São Paulo, Sao Paulo, SP Brazil; 5 Departamento de Cardiopneumologia, Instituto do Coração, InCor-HCFMUSP, Universidade de São Paulo, Sao Paulo, SP Brazil; 6 Laboratório de Investigação Médica- LIM 61, Serviço de Cirurgia Torácica, Faculdade de Medicina FMUSP, Universidade de Sao Paulo, Sao Paulo, SP Brazil; 7 Hospital Sírio-Libanês, Serviço de Reabilitação, Sao Paulo, SP Brazil; 8 Laboratório de Terapêutica Experimental (LIM-20), Faculdade de Medicina da USP (FMUSP), Universidade de Sao Paulo, Sao Paulo, Brazil

**Keywords:** Cleaning workers, Work-related asthma, Work-related rhinitis, Respiratory symptoms, Nasal swab

## Abstract

**Background:**

Cleaning workers represent a significant proportion of the active population worldwide, with poor remuneration, particularly in developing countries. Despite this, they remain a relatively poorly studied occupational group. They are constantly exposed to agents that can cause symptoms and respiratory problems. This study aimed to evaluate upper airway inflammation in professional cleaning workers in three different occupational settings by comparing nasal cytology inflammation and clinical profiles.

**Methods:**

We performed a cross-sectional study on the prevalence of upper airway inflammation and symptoms of asthma/rhinitis related to cleaning work, according to workplace. A total of 167 participants were divided into four groups: hospital, university, housekeeper and control. A nasal swab was collected for upper airway inflammation evaluation. Clinical profiles and respiratory symptom employee evaluations were performed using specific questionnaires (European Community Respiratory Health Survey—ECRS and the International Study of Asthma and Allergies in Childhood—ISAAC).

**Results:**

Cleaning workers showed increased neutrophils and lymphocytes; the hospital and university groups showed increased macrophages compared to the housekeeper and control groups. The hospital and housekeeper groups showed increased eosinophils when they performed cleaning services for up to one year and reported having more asthma symptoms than the control group. Cleaning workers showed increased rhinitis symptoms. The university group showed increased rhinitis symptoms aggravated by the workplace compared with the hospital and housekeeper groups. Cleaning workers showed an increased affirmative response when directly asked about rhinitis symptoms compared to the control group.

**Conclusions:**

Cleaning workers showed airway inflammation, asthma symptoms and rhinitis, regardless of the occupational environment to which they were exposed, as well as showed increased rhinitis and asthma symptoms. Hospital cleaning workers showed increased macrophages, lymphocytes and eosinophils compared to the others. The length of time spent performing cleaning work was not related to nasal inflammation or respiratory symptoms in this population. However, there were differences in workplaces. Registered on ClinicalTrials.gov. Trial registration number: NCT03311048. Registration date: 10.16.2017. Retrospectively registered.

**Supplementary Information:**

The online version contains supplementary material available at 10.1186/s12890-022-01949-5.

## Background

Occupational asthma (OA) is defined as asthma induced by occupational exposure, with or without pre-existing asthma [1]. The British Occupational Health Research Foundation (BOHRF) journal found that occupational factors represent one in six cases of asthma in working-age adults [2]. Occupational inhalants may cause asthma by sensitization, airway inflammation or irritant reflex [3].

Cleaning activities seem to represent an important risk, and an important portion of workers who have asthma symptoms related to cleaning materials have a pattern of bronchial reaction consistent with occupational asthma induced by sensitizers [4]. In the city of São Paulo, cleaning was the main occupation in terms of the number of cases of occupational asthma among women, and cleaning products were the most frequently reported agent between 1995 and 2000 [5].

Certain agents with unknown mechanisms or irritants may cause OA, and it can be mediated by IgE [6]. The mechanisms involved in asthma caused or exacerbated by cleaning agents are not clear. Both allergic and irritating mechanisms are implicated, but the irritant mechanism seems to be more relevant. The inflammatory response, particularly Th2, damages the epithelium of the airways due to continued exposure to the irritant [7].

High molecular weight (HMW) components (greater/equal 5000 Daltons) induce asthma through the production of specific IgE antibodies. Some low molecular weight (LMW) compounds (< 5000 Daltons), such as acid anhydrides and platinum salts, induce specific IgE antibodies by combining with a body protein [8]. Preformed and newly formed inflammatory mediators are released, and they orchestrate the inflammatory process [9]. OA induced by IgE-dependent agents is similar to allergic asthma, which is unrelated to work.

Both LMW and HMW were also identified as causes of occupational rhinitis (OR). The immunologic mechanisms involved in OR are different depending on the type of exposure [10].

OR is strongly associated with OA. It is important to elucidate OR, since it shares common pathophysiological features and trigger factors with OA. A better understanding of the interactions between the upper and lower airways can provide a stronger comprehension of the clinical characteristics of rhinitis, as both OR and OA share many causal factors [11].

Symptoms of allergic OR may precede the onset of OA [12, 13]. The presence of atopy or allergic conditions, such as eczema or allergic rhinitis, increases the likelihood of a diagnosis of allergic asthma in patients who have respiratory symptoms [14].

Although cleaners represent a significant proportion of the working population worldwide, they remain a relatively understudied occupational group. Cleaning workers represent a significant proportion of the active population worldwide, with poor remuneration, particularly in developing countries. They are constantly exposed to agents that can cause symptoms and respiratory problems [15, 16].

There is evidence that occupational exposure to disinfectants and cleaning products is related to other chronic respiratory diseases, such as rhinitis and poor pulmonary function. The effects of workplace exposure to sanitizers and cleaning agents as a cause or exacerbation of asthma are already well described. The onset of asthma caused by exposure to these substances has not decreased, so there is a need to focus on prevention efforts [17].

However, this study aimed to evaluate upper airway inflammation in professional cleaning workers in three different occupational settings by comparing nasal cytology inflammation and clinical profiles.

## Methods

### Study design, ethics statement and trial registration

This was a cross-sectional study on the prevalence of upper airway inflammation and symptoms of asthma/rhinitis related to cleaning work, according to the place of employment, approved by the IAMSPE Research Ethics Committee (protocol number 1514913.3.0000.5463, approval 2.426.900) (S1), Trial registration: Clinical Trials NCT03311048 (https://clinicaltrials.gov/ct2/show/NCT03311048) (retrospectively registered). The STROBE cross-sectional reporting guidelines were used in this study (S2) [15].

### Study population

Recruitment and data collection took place from April 2015 to December 2017 in the city of Cacoal, State of Rondônia, Brazil, and included cleaning workers divided into four groups:

(A) Hospital: individuals working in hospital cleaning;
(B) University: individuals working in university cleaning;
(C) Housekeeper: individuals working in professional cleaning of people's homes; and
(D) Control: individuals working in offices (not cleaners).

Active smokers, pregnant women, infants, and individuals receiving ongoing treatment for airway disorders were excluded from the study. All cleaning and office workers from each selected location (university, hospital, housekeeper and control) were invited, and all those who agreed to participate in the study and who did not meet any exclusion criteria were included. The control group was composed of office workers who did not deal with cleaning products (not cleaners).

### Demographic and work characteristics

Cleaning workers from all experimental groups answered a questionnaire on demographic and work characteristics, in addition to the main substances used to clean.

### Questionnaires

The exposure data were collected by a questionnaire structured by the authors that contained the following data: name, date of birth, sex, place of work, function, hours of work per day and week, and cleaning products most commonly used on a day-to-day basis (Additional file 1).

Information on respiratory symptoms was collected using a translation of the European Community Respiratory Health Survey (ECRHS) for occupational diseases [18] translated and validated into Portuguese [19] and the International Study of Asthma and Allergies in Childhood (ISAAC) asthma and rhinitis modules [20]. The asthma module had previously been validated in Portuguese [21]. Additionally, information about symptom onset and cleaning-related airway symptoms was obtained.

### Nasal swab test

The nasal swab protocol has been described previously by Ronchetti et al. [22]. A sterile swab was used (Johnson & Johnson, Brazil). Twenty minutes after collection, the slides were stained using May-Grunwald-Giemsa for the identification of eosinophils, neutrophils, lymphocytes, macrophages and epithelial cells. Cells were analyzed using a Nikon E600 (Nikon, Canada) optical microscope with a magnification of 1000×. A total of 200 cells were counted on two slides [22].

### Statistical analysis

Statistical analysis was performed using the Kruskal–Wallis test and the Dunn test for comparisons between groups. To evaluate the association between the qualitative variables, the chi-square test was used. The statistical software SigmaPlot 12.0 (Systat Software, San Jose, CA) and SPSS 21.0 (IBM, USA) were used for the analyses. The confidence interval was 95% (*p* < 0.05).

## Results

### Study population

**Fig. 1:**
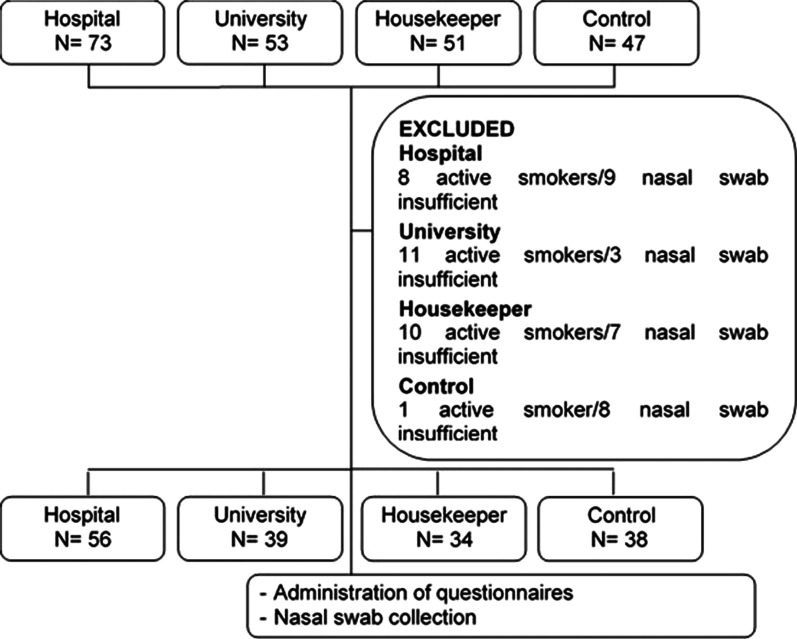
Study design

A total of 167 workers were recruited to participate in this study: 56 hospital cleaners, 29 university cleaners, 34 housekeepers and 39 office workers. Nine patients were excluded from the study because they met the exclusion criteria (Fig. 1).

### Demographic and work characteristics

The majority of the participants were female (84.4%), and most of the subjects worked full time (Table 1A and B). The cleaning products most used were bleaches, disinfectants, alcohol, detergents and others (Table 1C).

**Table 1:** Baseline characteristics and main products or substances by job location

	Hospital		University		Housekeeper		Control	
	N	%	N	%	N	%	N	%
*A. Demographic characteristics*								
Age (year) (mean ± SD)	36.6 (8.4)		36.8 (9.5)		37.5 (11.0)		27.6 (6.3)	
Sex								
Male	12	21.4	5	12.8	1	2.9	8	21.1
Female	44	78.6	34	87.2	33	97.1	30	78.9
	56	33.5	39	23.3	34	20.3	38	22.7
*B. Job characteristics*								
Work shift								
Day	24	21.6	27	24.3	22	19.8	38	34.2
Night	1	50.0	0	0.0	1	50.0	0	0.0
Varied	31	57.4	12	22.2	11	20.4	0	0.0
Hours worked per week								
40 (full-time)	52	33.1	39	24.8	28	17.8	38	24.2
36 (part-time)	4	40.0	0	0.0	6	60.0	0	0.0
*C. Main substances used*							Total	
Hypochlorite	28	38.3	20	27.4	25	34.2	73	100
Multipurpose cleaner	16	45.7	9	25.7	10	28.6	35	100
Formalin	2	22.2	6	66.6	1	11.1	9	100
Disinfectants	3	42.8	4	57.1	0	0.0	7	100
Washing powder	1	33.8	2	66.6	1	33.3	3	100
Liquid soap	0	0.0	1	50.0	1	50.0	2	100
Acid descaling detergent	0	0.0	1	2.5	0	0.0	0	0.0
Softener	0	0.0	0	0.0	1	2.9	0	0.0

*SD* Standard deviation

The most common work practices reported were scrubbing; washing; polishing; waxing; cleaning of carpets, vases, windows and kitchens; dusting; carpet tapping; using the washing machine; and hand washing (Table 2).

**Table 2:** Main work tasks performed by cleaning workers

Activity	Frequency	Hospital		University		Housekeeper		Control	
		N	%	N	%	N	%	N	%
Dusting, carpet cleaning	Daily	14	26.4	24	45.3	15	28.3	0	0.0
	No	40	38.1	15	14.3	12	11.4	38	36.2
	Fortnightly	1	25.0	0	0.0	3	75.0	0	0.0
	2× week	1	20.0	0	0.0	4	80.0	0	0.0
Scrubbing, washing	Daily	50	46.3	35	32.4	23	21.3	0	0.0
	No	6	11.3	4	7.5	5	9.4	38	71.7
	Fortnightly	0	0.0	0	0.0	1	100.0	0	0.0
	2× week	0	0.0	0	0.0	1	100.0	0	0.0
Cleaning of sinks and sanitary ware	Daily	23	47.7	31	27.9	27	24.3	0	0.0
	No	3	5.7	8	15.1	4	7.5	38	71.7
	Fortnightly	0	0.0	0	0.0	3	100.0	0	0.0
	2× week	0	0.0	0	0.0	0	0.0	0	0.0
Polishing, waxing	Daily	6	17.1	26	74.3	3	8.6	0	0.0
	No	48	37.8	13	10.2	28	22.0	38	29.9
	Fortnightly	2	40.0	0	0.0	3	60.0	0	0.0
	2× week	0	0.0	0	0.0	0	0.0	0	0.0
Window cleaning	Daily	42	49.4	26	30.6	17	20.0	0	0.0
	No	5	8.1	13	21.0	6	9.7	38	61.3
	Fortnightly	8	57.1	0	0.0	6	42.9	0	0.0
	2× week	1	16.7	0	0.0	5	83.3	0	83.3
Kitchen cleaning	Daily	16	43.2	12	32.4	9	24.3	0	24.3
	No	39	32.2	26	21.5	18	14.9	38	31.4
	Fortnightly	0	0.0	0	0.0	0	0.0	0	0.0
	2× week	1	11.1	1	11.1	7	77.8	0	0.0
Handwashing (clothing)	Daily	3	33.3	5	55.6	1	11.1	0	0.0
	No	52	34.2	34	22.4	28	18.4	38	25.0
	Fortnightly	1	20.0	0	0.0	4	80.0	0	0.0
	2× week	0	0.0	0	0.0	1	100.0	0	0.0
Machine washing (clothing)	Daily	9	37.5	3	12.5	12	50.0	0	0.0
	No	46	35.4	35	26.9	12	8.5	38	29.2
	Fortnightly	0	0.0	0	0.0	1	100.0	0	0.0
	2× week	1	8.3	1	8.3	10	83.3	0	0.0

According to the report of cleaning workers, hypochlorite, multipurpose products, powder, formaldehyde, disinfectants, laundry detergent and liquid soap were the main products used that were related to respiratory symptoms (Table 3).

**Table 3:** Main substances and products used and respiratory symptoms related by the cleaning workers as causes of respiratory symptoms

Study group/product	Hospital	University	Housekeeper	*p* value
Hypochlorite	N (%)	N (%)	N (%)	
Yes	28 (52.8)	20 (37.7)	25 (49.0)	0.270
No	25 (47.2)	33 (62.3)	26 (51.0)	
Multipurpose cleaner				
Yes	16 (45.7)	9 (25.7)	10 (28.6)	0.225
No	37 (30.3)	44 (36.1)	41 (33.6)	
Formalin				
Yes	2 (22.2)	6 (66.7)	1 (11.1)	0.092
No	51 (34.5)	47 (31.8)	50 (33**.**8)	
Disinfectants				
Yes	3 (42.9)	4 (57.1)	0 (0.0)	0.154
No	50 (33.3)	49 (32.7)	51 (34.0)	
Washing powder				
Yes	1 (25.0)	2 (50.0)	1 (25,0)	0.785
No	52 (34.0)	51 (33**.**3)	50 (32**.**7)	
Liquid soap				
Yes	0 (0.0)	1 (50.0)	1 (50.0)	0.596
No	53 (34.2)	52 (33.5)	50 (32.3)	
Acid descaling detergent				
Yes	0 (0,0)	1 (100.0)	0 (0.0)	0.373
No	53 (34.0)	52 (33.3)	51 (32.7)	
Acid				
Yes	0 (0.0)	1 (100.0)	0 (0.0)	0.373
No	53 (34.0)	52 (33.3)	51 (32.7)	
Softener				
Yes	0 (0.0)	0 (0.0)	1 (100.0)	0.351
No	53 (34.0)	53 (34.0)	50 (32.1)	

Yes: means that the product is used or that the cleaning worker has contact with the substance. No: means that the participant has no contact with the said product or substance

### Nasal swab test

**Fig. 2:**
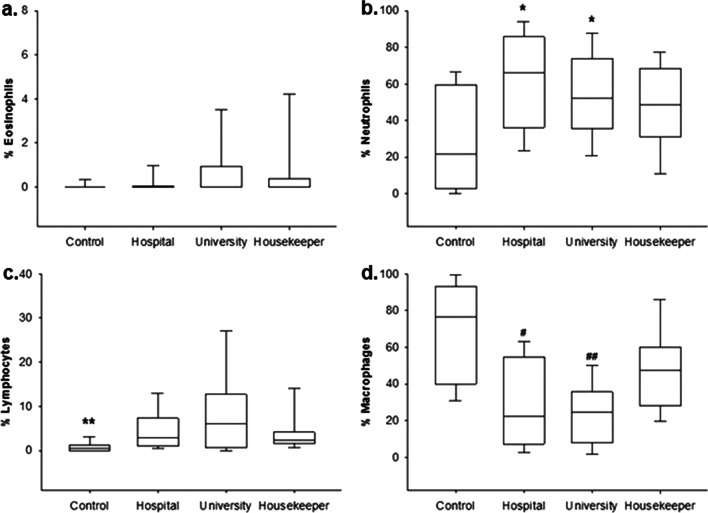
Nasal swab inflammatory cell percentage as workplace (%). **a** Eosinophils: no significant difference. **b** Neutrophils: The hospital and university groups showed differences compared to the control group (*p ≤ 0.001). **c** Lymphocytes: The control group showed differences compared to the others (**p ≤ 0.001). **d** Macrophages: The hospital and university groups showed differences compared to the housekeeper and control groups (^#, ##^*p* ≤ 0.001)

In the analysis of the cellularity in the nasal swab, it was not possible to observe a difference in relation to the percentage of eosinophils among the groups (Fig. 2a). A higher percentage of neutrophils was observed in the hospital and university groups when compared to that in the control group (*p* ≤ 0.001) (Fig. 2b). The percentage of lymphocytes in the control group was lower than that in the other groups (*p* ≤ 0.001) (Fig. 2c). The percentage of macrophages in the hospital and university groups was significantly lower than that in the housekeeper and control groups (*p* ≤ 0.001) (Fig. 2d).

**Fig. 3:**
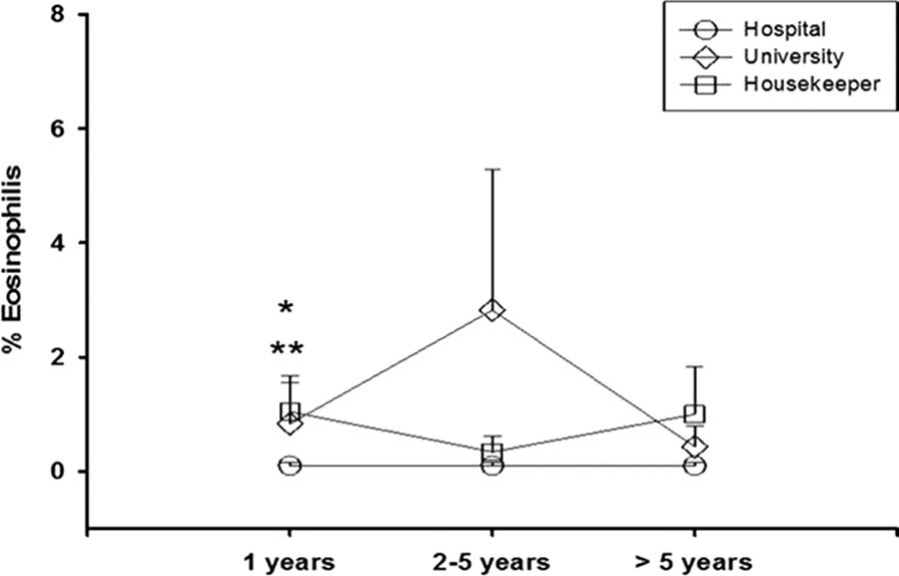
Nasal swab eosinophil percentage as years worked in cleaning services. *p = 0.001 compared to the hospital group; **p = 0.001 compared to 1 year of housekeeping with 2–5 years of housekeeping

The cellularity in the nasal swab of the cleaning workers was also compared according to the length of performance of the labor activity: up to 1 year, 2–5 years and > 5 years. A higher percentage of eosinophils was observed among the housekeeper group in relation to the hospital group for the workers with up to a year of cleaning service (*p* < 0.05) (Fig. 3 and Table 4).

**Table 4:** Nasal swab neutrophils and eosinophils as years in cleaning work, by job location

Job tenure (years)	Hospital group		University group		Housekeeper group		*P*	
	Eos (%)	Neut (%)	Eos (%)	Neut (%)	Eos (%)	Neut (%)	Eos	Neut
	(Mean ± SD)		(Mean ± SD)		(Mean ± SD)			
1	0.09 ± 0.06	55.59 ± 7.65	0.83 ± 0.72	37.16 ± 15.73	1.04 ± 0.62*	43.82 ± 7.82	0.05	NS
2–5	0.09 ± 0.05	63.07 ± 8.08	2.82 ± 2.45	47.62 ± 8.13	0.34 ± 0.28	43.14 ± 7.00	NS	NS
> 5	0.09 ± 0.05	36.07 ± 9.59	0.43 ± 0.36	43.25 ± 9.74	1.00 ± 0.83	33.40 ± 8.24	NS	NS

NS: not significant. “*p*”: value significant

*p < 0.05 Housekeeper compared to hospital

### Questionnaires

We observed that the housekeeper group had a lower frequency of the symptom "accompanying other people's walking" than the other groups (*p* < 0.01) (Table 5A). In relation to the other symptoms, there was no difference between cleaning workers, regardless of their workplace (Table 5A).

**Table 5:** Primarily reported airway symptoms, asthma and rhinitis, as well as reported symptoms related to or aggravated by job

	Hospital	University	Housekeeper	Control	*P*
	N (%)	N (%)	N (%)	N (%)	
*A. Symptom*					
Wheezing	16 (39.0)	11 (26.8)	14 (34.1)	0 (0.0)*	< 0.05
Wheezing (not cold)	9 (45.0)	3 (15.0)	8 (40.0)	0 (0.0)*	< 0.05
Chest tightness (night)	12 (34.3)	8 (22,9)	15 (42.9)	0 (0.0)*	< 0.05
Shortness of breath	10 (37.0)	8 (29,6)	9 (33.3)	0 (0.0)*	< 0.05
Coughing fit (night)	14 (35.0)	14 (35.0)	12 (30.0)	0 (0.0)*	< 0.05
Asthma attack	2 (22.2)	4 (44.4)	3 (33.3)	0 (0.0)	0.16
Cough upon waking	7 (38.9)	2 (11.1)	9 (50.0)	0 (0.0)*	< 0.05
Breathlessness	20 (43.5)	13 (28.3)	13 (28.3)	0 (0.0)*	< 0.05
Walking alongside other people	45 (34.4)	36 (27.5)	13 (9.9)**	37 (28.2)	< 0.01
Stop to rest while walking	5 (41.7)	3 (25.0)	4 (33.3)	0 (0.0)	0.23
Sneezing, runny nose	22 (45.8)	12 (25.0)	14 (29.2)	0 (0.0)*	< 0.05
Rhinitis (reported)	16 (59.3)	6 (22.2)	5 (18.5)	0 (0.0)*	< 0.05
*B. Asthma symptoms related to or aggravated by job*					
WRA	6 (75.0)^+^	1 (12.5)	1 (12.5)	–	0.06
WAA	2 (25.0)	2 (25.0)	4 (50.0)	–	0.29
*C. Rhinitis symptoms related to or aggravated by job*					
WRR	11 (33.3)	11 (33.3)	11 (33.3)	–	0.16
WAR	10 (66.7)	0 (0.0)^++^	5 (33.5)	–	< 0.01

^*^p < 0.05: Control group compared to the others. **p < 0.01: Housekeeper group compared to the others for “walking alongside other people.” ^**+**^*p* = 0.06 Hospital group compared to the university and housekeeper groups. ^++^*p* < 0.01 University group compared to the hospital and housekeeper groups. Abbreviations: WRA: work-related asthma. WAA: work-aggravated asthma. WRR: work-related rhinitis. WAR: work-aggravated rhinitis. NS: not significant. “*p*”: value significant

Cleaning workers were questioned about the onset and worsening of asthma or rhinitis symptoms. If the symptoms of asthma appeared only after the beginning of their cleaning work, the symptoms were considered to indicate "work-related asthma" (WRA); if these symptoms already existed and worsened after participants started their cleaning work, the symptoms were referred to as "work-aggravated asthma" (WAA). The same was considered for rhinitis symptoms, classified as "work-related rhinitis" (WRR) if the onset occurred after participants began their cleaning work and "work-aggravated rhinitis" (WAR) if they already existed and worsened after performing professional cleaning (Table 5B and C).

Thus, the hospital group reported more symptoms in relation to WRA than did the university and housekeeper groups (*p* = 0.06). In relation to WAA, there was no difference among the groups (Table 5B); the same occurred in relation to WRR (Table 5C). However, in relation to WAR, the university group reported more symptoms than did the hospital and the housekeeper groups (*p* ≤ 0.001) (Table 5C).

## Discussion

This study showed a higher percentage of neutrophils in the hospital and university groups when compared to that in the control group and a decreased percentage of macrophages in the housekeeper and control groups when compared to that in the hospital and university groups. The percentage of lymphocytes increased in the university, hospital, housekeeper and control groups when compared to that in the hospital and university groups. A lower percentage of eosinophils in the nasal mucosa was observed in the housekeeper group when compared to that in the hospital group among the workers with up to 1 year of cleaning service experience.

A study performed with a population of bakers reported that they complained more about symptoms of the upper airways than of the lower airways. They showed inflammation of the nasal mucosa confirmed by nasal cytology, while the majority of the subjects exhibited neutrophilic rhinitis. This study supposes that long-term exposure to occupational bakery dust may result in the development of sensitization to job-related allergens and status of minimal nasal inflammation and lower respiratory tract inflammation [12].

As exemplified in a study on the inflammatory process in the airways of patients with allergic and non-allergic asthma, eosinophils play an important role in the allergic inflammatory process. In the airways, inflammation is the result of complex interactions between inhaled allergens, immune cells and structural cells, such as epithelial cells, endothelial cells and fibroblasts. Neutrophils also have an important role in inflammation associated with asthma, including in the severe asthma phenotype where neutrophilic inflammation predominates [23]. In addition, Rank et al., in 2016, demonstrated a strong association in a given patient between the concentrations of eosinophilic nasal and pharyngeal peroxidase and the percentage of eosinophil-induced sputum [24].

The incidence of occupational respiratory diseases is underestimated both by the difficulty of diagnostic confirmation and by the reluctance of the worker, who often does not seek medical care to confirm the diagnosis for fear of losing his or her job. Even so, the growing increase observed in the incidence of occupational diseases has drawn increasing health-related attention to the work environment [25].

Symptomatology in work-related respiratory disease is an extremely important factor to be considered by health professionals [6]. Our findings revealed that major complaints, such as wheezing, chest tightness, shortness of breath, coughing, sneezing, runny nose and rhinitis, were present only in the cleaning worker groups. Notably, water-soluble cleaning products can irritate the upper airways [26].

Our study also revealed that the prevalence of asthma was higher not only among hospital employees or housekeepers but also among workers from all cleaning groups, showing that professional cleaning activities could be related to work-related asthma, since no professional in the control group was identified as having asthma. These results are consistent with those of many other studies [7, 27, 28].

The performance of cleaning services as a risk factor for developing asthma was described in a study by Karjalainen et al. [29] that followed up with cleaning and office professionals for 12 years, noting that the odds ratio for asthma among cleaners was 1.5-fold (CI of 1.43–1.57) higher than that among professionals working in offices.

The number of women in our research was higher than the number of men in all groups of workers studied. Maçãira et al. [21] showed the importance of studying this population, given that the respiratory morbidity in internal cleaning workers in the metropolitan region of São Paulo reflected twice the length of exposure to risk factors for respiratory diseases, and the prevalence of inhaled accidents in women was three times higher than that in men.

Exposure to cleaning products is another known risk factor, and several studies have shown that an individual's susceptibility should also be taken into account; although the work environment is the same for different individuals, some develop respiratory and other diseases [28, 30].

Atopy and smoking are some of the characteristics described in the literature as factors that contribute to this scenario [31]. It should be emphasized that, in this protocol, all subjects were excluded from active tobacco use, with only a few former smokers, representing less than 2.5% of the population studied in our study (4 individuals).

It is known that nasal and ocular symptoms are more important in the presence of high molecular weight agents than in the presence of lower molecular weight agents. Among cleaning workers, the use of low molecular weight products is more common, and many of these products are irritants [21].

Rhinitis of allergic origin may or may not induce the onset of asthma in people who have never had pulmonary diseases [32], such as in the study by Bauchau and Durham [33], who demonstrated that allergic rhinitis was more prevalent among cleaning professionals than in the general population. These data corroborate our findings, which demonstrated that individuals from the three groups of cleaning workers had more respiratory symptoms than office workers.

The data found in our study on rhinitis demonstrated that both the presence of rhinitis-defining symptoms and self-reported rhinitis were greater among cleaners than among office workers. However, there was no relationship between these variables and the percentage of eosinophils or neutrophils. The absence of this relationship can be justified by the lack of specificity of the symptom questionnaires for the diagnosis of work-related rhinitis, since these are subjective and since we observed that there was an increase in the percentages of these cells.

Thus, despite the absence of clinical tests to prove the existence of asthma and rhinitis in our population, the high specificity and sensitivity of the ISAAC asthma module [21] scores justify the use of this instrument.

Folletti et al. [34] systematically reviewed 24 studies addressing the relationship between cleaning work and the risk of asthma and rhinitis and rephrased that an increased risk of asthma or rhinitis was demonstrated in 79% of the included epidemiological studies. Confirmation of this information was made mainly by objective tests, such as bronchial hyperreactivity or airflow obstruction. The specific causes associated with the onset of asthma and rhinitis were the level of exposure to cleaning products, sprays, hypochlorite, ammonia, product mix and specific work tasks.

In our study, the time of service and the hours worked were crossed with the information of respiratory symptoms. We identified that the service time between the groups studied was the same. After this, we evaluated the differences among the three service time ranges (up to 1 year, 2–5 years and > 5 years) and asthma and rhinitis symptoms.

In the first evaluation, there was no difference in asthma symptoms among workers, according to the length of service intervals. However, for rhinitis symptoms, university workers with less than 1 year of experience had fewer symptoms than the others did. These results differ from those found by Slavin [35], who reported that the incidence of rhinoconjunctival symptoms with an occupational etiology was higher in the first 12–20 months of professional activity, with a progressive increase when the exposure was continuous for 24 months.

Having to work over 20 h weekly was more common among hospital workers with asthma symptoms. We believe that longer work time is directly linked to the occurrence of more symptoms; the same result was found when symptoms of rhinitis were evaluated and rhinitis was confirmed, with a higher number of working hours evidenced in hospital workers. However, the alterations found in our population did not express nasal cellularity. In the analysis of the correlation between the time of service and the percentage of eosinophils and neutrophils, no significant difference was found.

When the type of analysis was adjusted for comparison rather than correlation, we identified that hospital employees and day laborers with less than 1 year of professional performance differed from each other concerning the percentage of eosinophils but not neutrophils. Hospital workers had lower percentages of eosinophils, followed by housekeepers, and finally, university workers had higher percentages of eosinophils. These results, together with the significant values found for the duration of symptoms in professionals working in hospitals, convey that this professional category is more affected in both symptomatology and cellularity.

Considering the results of eosinophil counts in subjects with rhinitis from previous studies [36, 37] and comparing these values to those found in our study, it can be verified that the rate of eosinophils does not correspond to that expected for individuals with rhinitis in all groups studied (i.e., > 5%). However, the percentages of the three groups of cleaning workers were higher than those found in the control group (hospital: 0.7 ± 2.4; university: 1.2 ± 3.4; housekeeper: 0.7 ± 1.7; control: 0.05 ± 0.1; percentages of eosinophils).

This same justification is exemplified in the study by Pal et al. [38], which showed that the difference in the mean eosinophil counts of patients with allergic rhinitis and controls was statistically significant, and a nasal smear eosinophil count > 0.3 per high-power field (HPF) had 100% specificity and 100% positive predictive value for allergic rhinitis. Asthma was associated with allergic rhinitis in 40% of patients; an association was not found between nasal smear eosinophil count and the symptoms, duration, type, and severity of allergic rhinitis or coexistent asthma. These authors concluded that an eosinophil count > 0.3 per HPF in nasal smears is a highly specific criterion for the diagnosis of allergic rhinitis. When grouping and comparing all cleaners who answered “yes” to the question of the presence of rhinitis and asthma, regardless of the length of service, these responses differed significantly for those who answered negatively to the same questions. As found in this study, evidence of changes in cell rates related to work-related rhinitis has been described in several other studies [39, 40].

In a large random population study, chronic rhinosinusitis was substantially related with exposure to various airborne irritants and sensitizers in the labour environment, such as cleaning agents, paper dust, metal dust, animals, moisture/mold/mildew and physically strenuous work [41].

Lovato et al. [42] compared a group of carpenters with nonexposed individuals to determine whether exposure to wood dust was correlated with specific patterns of inflammatory or infectious rhinitis. The authors identified that carpenters reported significantly more nasal symptoms than the control group (*p* = 0.0007). The nasal smears of the carpenter group contained significantly more neutrophils (*p* < 0.00001) and lymphocytes (*p* = 0.02) than did those of the control group, indicating that nasal cytology was able to reveal chronic inflammatory rhinitis in a significant proportion of carpenters, highlighting the potential of the technique in the screening of this pathology.

Gelardi et al. [43] also agree with the potential of the nasal cytology technique and emphasize that it deserves its place in the arsenal of diagnostic techniques for chronic rhinitis because it is an easy, reliable, and inexpensive method in the absence of other diagnostic tools. It can be used to identify and measuring the cell population inside the nasal mucosa quickly; help to better distinguish various pathological conditions; and measure the effects of several stimuli, such as allergens, infections, irritants, physical substances, compound, or treatments.

The present study presents some limitations. First, we can mention the self-selection bias. Cleaning workers in the hospital, university and housekeeper groups were selected for the study to evaluate individuals in different workplaces. It is possible that these workers volunteered more easily because they believe they have some comorbidity, and if this is true, it may have generated bias. Regarding the healthy worker effect, we found that the control group was composed of healthy volunteers who worked in an office environment, so we sought to select people who were not exposed to cleaning products. Although we consider that this selection may have created another bias, it was expected that these individuals would not present with respiratory symptoms. We thought it was important to have a control group because there was no way to evaluate the exposure of cleaning workers outside the work environment; thus, when compared with unexposed workers, we inferred that the function of cleaning workers could harm the airways and cause respiratory symptoms.

The control group was on average at least 10 years younger than the other groups, and this fact may have generated another bias; however, cleaning workers and even younger workers also reported respiratory symptoms.

There was only an association of asthma symptoms or rhinitis in the housekeeper, hospital and university groups, and there was no association with the control group because there was no report of related symptoms in this group. Thus, multivariate analysis was not performed, because the predictive variables were not significant; that is, they were not associated with the outcome.

We can also cite that no specific equipment was used to determine the levels of chlorine or other air components in the exposure atmosphere. Last, the patients also did not undergo a clinical evaluation, and the classification of symptoms was made according to the answers to the questionnaires.

As for the strengths of this study, we investigated associations between asthma and occupational exposure and several cleaning agents. We believe that further studies revealing the implicit risks of using cleaning products are of great value for this profession, which has been growing in recent years in large cities. We emphasize the importance of continued investigation. In addition, future studies are necessary to clarify the risk of the use of these chemical agents, with the goal of greater adherence to the use of personal protective equipment, as well as preventive measures to minimize the risks to cleaning employees, regardless of where they work.

## Conclusions

Cleaning workers had upper airway inflammation, as well as symptoms of asthma and rhinitis, regardless of the workplace to which they were exposed. Although previous studies have shown that cumulative cleaning time is an asthma-related risk factor, cleaning work time was not related to nasal inflammation and respiratory symptoms in this population.

## Supplementary Information

**Additional file 1.** Sociodemographic questionnaire.

## Data Availability

All relevant data are within the paper, and the main data can also be accessed at https://clinicaltrials.gov/ct2/show/results/NCT03311048. Trial registration: NCT03311048, registration date: 10.16.2017, retrospectively registered. URL: www.clinicaltrials.gov. Any additional data can be requested from the corresponding author or the last author.

## Abbreviations

BOHRF: British Occupational Health Research Foundation
EOS: Eosinophils
ECRHS: European Community Respiratory Health Survey
HMW: High molecular weight
IgE: Immunoglobulin E
ISAAC: International Study of Asthma and Allergies in Childhood
LMW: Low molecular weight
NEUT: Neutrophils
OA: Occupational asthma
OR: Occupational rhinitis
WAA: Work-aggravated asthma
WAR: Work-aggravated rhinitis
WRA: Work-related asthma
WRR: Work-related rhinitis
