# Supplementary material for: Airway inflammatory profile among cleaning workers from different workplaces
Source: BMC Pulm Med. 2022 Apr 29;22:170. doi: 10.1186/s12890-022-01949-5 (PMC9052628; doi:10.1186/s12890-022-01949-5)
Supplement: Supplementary file 1 — Additional file 1. Sociodemographic questionnaire. [file 12890_2022_1949_MOESM1_ESM.pdf]

SOCIODEMOGRAPHIC QUESTIONNAIRE

Name:____

Address:____

Phone:____

Date of birth:_____________Sex:________________________________

Place of work:____

Function:____

Hours of work per day:________Hours of work per week:_________

Cleaning products most commonly used on a day-to-day basis:

____

____

____

____

Comments: ____

____

____

____
